# Supplementary material for: Rethinking input complexity in transformer-based clinical prediction: implications for feature dimensionality and sequence length in longitudinal electronic health record data
Source: JAMIA Open. 2026 Sep 30;9(5):ooag193. doi: 10.1093/jamiaopen/ooag193 (PMC13625019; doi:10.1093/jamiaopen/ooag193)
Supplement: Supplementary file 1 [file ooag193_supplementary_data.docx]

**Supplementary Material**

**Rethinking Input Complexity in Transformer-Based Clinical Prediction: Implications for Feature Dimensionality and Sequence Length in Longitudinal EHR Data**

**Table E1. Feature pool and feature subsets under each dimensionality-reduction strategy**

| **Feature** | **Feature pool/input space** | **Feature subsets** | | | | | | |
| --- | --- | --- | --- | --- | --- | --- | --- | --- |
|  |  | **IG** | | | **AUC** | | | **Clinical (pre-specified)** |
| **Number of features** | **80** | **10** | **20** | **40** | **10** | **20** | **40** | **20** |
| Age | x | x | x | x | x | x | x | x |
| Sex | x | x | x | x |  | x | x | x |
| Race/ethnicity | x | x | x | x |  |  | x | x |
| Body mass index | x |  |  | x | x | x | x | x |
| Smoking | x |  |  | x |  |  |  | x |
| Asthma action plan | x |  | x | x |  |  | x |  |
| Exercise minutes per week | x | x | x | x | x | x | x |  |
| Antacids | x |  |  | x |  |  | x |  |
| Antianginal agents | x |  |  |  |  |  |  |  |
| Antianxiety agents | x |  |  |  |  |  | x |  |
| Antiarrythmics | x |  |  |  |  |  |  |  |
| Antibacterial/microbial agents | x |  | x | x | x | x | x | x |
| Anticoagulants | x |  |  |  |  |  | x |  |
| Antidepressants | x |  |  |  |  | x | x | x |
| Antidiabetics | x |  |  |  |  |  | x | x |
| Antifungal agents | x |  | x | x |  |  |  |  |
| Antihyperlipidemics | x |  |  |  |  |  | x |  |
| Antihypertensive agents | x |  |  |  |  | x | x |  |
| Antiobesity agents | x |  |  | x |  |  |  |  |
| Antipsychotics | x |  |  |  |  |  |  |  |
| Antiseptics and disinfectants | x |  | x | x |  |  |  |  |
| Antiviral agents | x |  |  | x |  |  |  |  |
| Asthma agents | x |  |  | x |  |  |  |  |
| Asthma controllers | x |  |  | x | x | x | x | x |
| Beta blockers | x |  |  |  |  |  | x |  |
| Biologics | x |  |  | x |  |  |  |  |
| Calcium channel blockers | x |  |  |  |  |  |  | x |
| Diuretics | x |  |  |  |  |  | x |  |
| Glimepiride | x |  |  |  |  |  |  |  |
| Glipizide | x |  |  |  |  |  |  |  |
| Glyburide | x |  |  |  |  |  |  |  |
| Hyponotics | x |  |  |  |  |  |  |  |
| Inhaled corticosteroid | x |  | x | x | x | x | x |  |
| Inhaled corticosteroid / Long-acting β-agonist | x |  |  | x |  | x | x |  |
| Insulin | x |  |  |  |  |  |  |  |
| Leukotriene modifier | x |  |  | x |  |  | x |  |
| Long-acting muscarinic antagonist | x |  |  | x |  |  |  |  |
| Long-acting β-agonist | x |  |  | x |  |  |  |  |
| Long-acting β-agonist/ Long-acting muscarinic antagonist | x |  |  |  |  |  |  |  |
| Metformin | x |  |  |  |  |  |  |  |
| Proton pump inhibitor | x |  |  |  | x | x | x | x |
| Short-acting muscarinic antagonist | x |  | x | x |  | x | x |  |
| Short-acting β-agonist | x | x | x | x | x | x | x | x |
| Short-acting β-agonist/ Short-acting muscarinic antagonist | x |  |  | x |  |  |  |  |
| Somatostatic agents | x |  |  |  |  |  |  |  |
| Sulfonylureas | x |  |  |  |  |  |  |  |
| Systemic corticosteroid | x | x | x | x | x | x | x | x |
| Theophylline | x |  |  |  |  |  |  |  |
| Ulcer drugs | x |  |  |  |  | x | x |  |
| Influenza vaccination | x |  | x | x |  | x | x |  |
| Allergic rhinitis | x |  |  | x |  |  | x | x |
| Anemia | x |  |  |  |  |  | x |  |
| Anxiety | x |  |  |  |  |  | x | x |
| Anxiety and phobic disorders | x |  |  | x |  |  |  |  |
| Anxiety disorders | x |  |  |  |  |  |  |  |
| Anxiety obsessive-compulsive disorders | x |  |  |  |  |  |  |  |
| Asthma | x | x | x | x |  |  |  |  |
| Atopic dermatitis | x |  | x | x |  |  |  |  |
| Cerebrovascular disease | x |  |  |  |  |  |  |  |
| Chronic rhinitis | x |  |  | x |  |  | x |  |
| Chronic sinusitus | x |  |  | x |  | x | x | x |
| Dementia | x |  |  |  |  |  |  |  |
| Depression | x |  |  |  |  |  | x |  |
| Diabetes | x |  |  |  |  |  | x |  |
| Gastroesophageal reflux disease | x |  |  | x |  |  | x |  |
| Heart disease | x |  |  |  |  |  |  |  |
| Hyperlipidemia | x |  |  |  |  | x | x |  |
| Hypertension | x |  |  |  |  | x | x | x |
| Nasal polyp | x |  |  | x |  |  |  | x |
| Pneumonia/respiratory infections | x |  | x | x |  |  | x |  |
| Post-nasal drip | x | x | x | x |  |  |  |  |
| Sleep disorders | x |  |  |  |  |  | x |  |
| Sleep disorders - insomnia | x |  |  |  |  |  |  |  |
| Sleep disorders - others | x |  |  | x |  |  |  |  |
| Sleep disorders - sleep apnea | x |  |  |  |  |  |  |  |
| Eosinophil counts | x | x | x | x |  |  | x | x |
| Aeroallergen test | x |  |  |  |  |  |  |  |
| Positive aeroallergen test | x |  | x | x |  |  |  |  |
| H Pylori | x |  |  |  |  |  |  |  |
| Asthma ED or UC visit | x | x | x | x | x | x | x | x |

Columns indicate whether each feature was included (×) in the corresponding feature set. The “All Features (80)” column represents the complete feature pool available for model input. All other columns (IG10–IG40, AUC10–AUC40, and Clinical 20) represent features subsets using the specified dimensionality-reduction strategies applied to training data only. Integrated Gradients (IG)–based and AUC-based feature subsets were derived algorithmically, whereas the Clinical 20 subset was prespecified based on domain expertise.

**Table E2A. Sensitivity analysis of training batch size on external-test discrimination**

External test AUROC across six cross-validation folds and batch sizes

| **Fold** | **Batch Size = 32** | **Batch Size = 64** | **Batch Size = 128** |
| --- | --- | --- | --- |
| Fold 1 | 0.8403 | 0.8404 | 0.8408 |
| Fold 2 | 0.8436 | 0.8445 | 0.8451 |
| Fold 3 | 0.8549 | 0.8561 | 0.8567 |
| Fold 4 | 0.8460 | 0.8467 | 0.8480 |
| Fold 5 | 0.8523 | 0.8540 | 0.8543 |
| Fold 6 | 0.8503 | 0.8509 | 0.8518 |
| **Average** | **0.8479 ± 0.0051** | **0.8488 ± 0.0054** | **0.8495 ± 0.0054** |

Values represent the area under the receiver operating characteristic curve (AUROC) evaluated on the external test dataset across six cross-validation folds. Results are shown for the Integrated Gradients–selected model using 20 features, with maximum sequence length set to the 80th percentile of the empirical sequence-length distribution (max_len = p80; corresponding to 133 input elements). Training batch size was varied (32, 64, and 128) while all other model architecture, input representation, and training parameters were held constant.

**Table E2B. Sensitivity analysis of training batch size on computational runtime**

Average training time (hours) across six cross-validation folds

| **Fold** | **Batch Size = 32** | **Batch Size = 64** | **Batch Size = 128** |
| --- | --- | --- | --- |
| Fold 1 | 1.3761 | 1.2494 | 1.2000 |
| Fold 2 | 1.3167 | 1.1886 | 1.1547 |
| Fold 3 | 1.2136 | 1.1097 | 1.0642 |
| Fold 4 | 1.4014 | 1.2672 | 1.2239 |
| Fold 5 | 1.3803 | 1.2444 | 1.2028 |
| Fold 6 | 1.2558 | 1.1486 | 1.1053 |
| **Average** | **1.3240 ± 0.0692** | **1.2013 ± 0.0575** | **1.1585 ± 0.0573** |

Values represent average total model training time (in hours) across six cross-validation folds. Results are shown for the Integrated Gradients–selected model using 20 features, with maximum sequence length set to the 80th percentile of the empirical sequence-length distribution (max_len = p80; corresponding to 133 input elements). Training batch size was varied (32, 64, and 128) while all other model architecture, feature representation, and training settings were held constant.

**Figure E1. Construction of transformer input sequences from longitudinal electronic health record (EHR) data.**

**(A)** Example of a patient's longitudinal EHR timeline during the 730-day look-back window preceding the index date (Day 0). For cases, Day 0 represents the acute asthma exacerbation (AAE) index date; for controls, Day 0 corresponds to the matched case's index date. Clinical events occur at irregular intervals and include diagnoses, medication dispensings, laboratory results, and healthcare encounters.

**(B)** Conversion of longitudinal EHR events into an ordered sequence of tokens. Each clinical concept is represented as an individual token, with multiple tokens permitted on the same calendar day. Static patient characteristics (e.g., age, sex, and race/ethnicity) are encoded separately as time-invariant tokens. An end-of-visit (END) token separates clinical events from the same encounter while preserving their chronological order. The outcome at Day 0 is not included in the input sequence.

**(C)** Final transformer input sequence consisting of static tokens followed by chronologically ordered longitudinal tokens. The ordered token sequence is converted into embeddings and processed by the transformer encoder to generate the patient representation used for case-control classification.

Abbreviations: AAE, acute asthma exacerbation; CLS, classification token; Dx, diagnosis; ED, emergency department; END, end-of-visit token; EHR, electronic health record; ICS, inhaled corticosteroid; Lab, laboratory test; Med, medication; SABA, short-acting β-agonist; SEP, separator token; UC, urgent care.

**Figure E2. Sensitivity of event capture to risk-selection threshold**

Cumulative event capture curves showing the fraction of observed asthma exacerbation events captured as progressively larger fractions of observations are selected based on predicted risk. Curves are shown for the full-feature reference model and representative reduced-feature models.

This analysis complements fixed-threshold evaluations by assessing the robustness of event concentration across a broader range of operational risk-selection thresholds.
